# Neonatal Invasive Candidiasis: Current Concepts

**DOI:** 10.1007/s12098-025-05593-9

**Published:** 2025-06-03

**Authors:** Laura Ferreras-Antolin, Anuradha Chowdhary, Adilia Warris

**Affiliations:** 1 Pediatric Infectious Diseases Unit, St George’s University Hospitals NHS Foundation Trust, London, UK; 2 Department of Biosciences, MRC Centre for Medical Mycology, Faculty of Health and Life Sciences, University of Exeter, Exeter, UK; 3 Medical Mycology Unit, Department of Microbiology, Vallabhbhai Patel Chest Institute, University of Delhi, Delhi, India; 4 National Reference Laboratory for Antimicrobial Resistance in Fungal Pathogens, Vallabhbhai Patel Chest Institute, University of Delhi, Delhi, India; 5 Department of Pediatric Infectious Diseases, Great Ormond Street Hospital, London, UK

**Keywords:** Neonates, Candidiasis, *Candida parapsilosis*, *Candida auris*, Outbreaks, Infection prevention and control

## Abstract

Neonatal invasive candidiasis (NIC) is an important cause of morbidity and mortality, especially in preterm and very low birthweight infants, with the incidence being inversely correlated with gestational age and birth weight. Epidemiological studies in high-income countries show *Candida albicans* and *Candida parapsilosis* being the commonest species causing NIC, while in low- and middle-income countries NIC is more often caused by non-*albicans Candida* species with higher rates of antifungal resistance. The increased incidence of fluconazole-resistant *C. parapsilosis* and multi-drug-resistant *Candida auris* causing NIC is of concern and challenges our management approaches. Identification of the causative *Candida* sp. and susceptibility testing needs to be optimised to guide targeted treatment. Infection prevention and control measures are crucial to prevent neonatal *Candida* colonisation and the development of NIC. In this concise review, authors will discuss the global changing epidemiology of NIC, the increase of antifungal resistant *Candida* species and outbreaks, current management approaches, and the value of infection prevention and control measures in the prevention of NIC.

## Introduction

Neonatal invasive candidiasis (NIC) is an important cause of morbidity and mortality, especially in preterm and very low birthweight infants with the incidence being inversely correlated with gestational age and birth weight [1]. These infants have several additional risk factors related to immaturity of the immune system and the management of critical illness, making them vulnerable to develop NIC [2]. Species of *Candida* which cause NIC are acquired either vertically, during vaginal delivery from a colonized mother, or horizontally from the neonatal intensive care unit (NICU) environment [1]. The latter scenario is frequently reported from low- and middle-income settings where compromised hospital practices may lead to sporadic outbreaks of NIC. The incidence of NIC varies across geographic areas and also between different centres within the same region. Epidemiological studies in high income countries (HICs) show *Candida albicans* and *Candida parapsilosis* being the commonest species causing NIC [3, 4]. An important aspect regarding the epidemiology of NIC in low- and middle-income countries (LMICs) is the high occurrence of antifungal-resistant *Candida* spp. infections in NICUs [5]. It is noteworthy that the challenges in controlling these infections in LMICs are multifactorial including compromised healthcare due to limited resources, high patient load in public sector hospitals, non-availability of mycology diagnostics, and sub-optimal adherence in implementing guidelines. In this review authors will discuss the global changing epidemiology of NIC with a special focus on India, the increase of antifungal resistant *Candida* species and outbreaks, current management approaches, and the value of infection prevention and control measures in the prevention of NIC.

## Changing Epidemiology

The global fungal epidemiology of neonatal candidiasis has dramatically changed over the last decade, characterized by an increase in antifungal resistant *Candida* species. The emergence of the multi-drug-resistant *Candida auris* and the increase in fluconazole resistant *Candida parapsilosis* isolates, are challenging the management of neonatal candidiasis. Till recently, neonatal candidiasis was predominantly caused by *C. albicans*, followed by *C. parapsilosis*, with both species being susceptible to fluconazole [3, 4]. An active population-based candidemia surveillance in 4 US metropolitan areas between 2009 and 2015 showed that among neonates, the annual incidence of candidemia cases decreased from 31.5 per 100 000 births in 2009 to between 10.7 and 11.8 per 100 000 births per year between 2012 and 2015 [6]. Prematurity was the most common underlying condition (78%) among neonatal cases. Nearly all of the premature neonates had a very low birth weight (1000–1500 g) or extremely low birth weight (< 1000 g). *Candida albicans* was the most common species among neonatal cases (68%) and *C. parapsilosis* was the most common non-*albicans Candida* (NAC) species [6]. A retrospective multicentre European study of the epidemiology and outcome of neonatal and pediatric candidemia analysed 1395 episodes of candidemia from 23 hospitals in 10 European countries. Of the 1395 episodes, 36.4% occurred in neonates and *C. albicans* (52.5%) and *C. parapsilosis* (28%) were the predominant species [3]. Also, a prospective, multicentre, surveillance study of candidemia in a pediatric population from 23 hospitals in 8 Latin America countries between 2008 and 2010 reported that the main species isolated in neonates were *C. albicans* (43.8%), *C. parapsilosis* (27.0%) and *Candida tropicalis* (14.6%) [4]. Over the last 5 y, reports from NICUs, especially in LMICs, show that *C. auris* is becoming a major cause, and in some regions the leading cause, of NIC [7–9]. Only for a minority of these *C. auris* isolates susceptibility testing was performed, showing fluconazole-resistance of 73–97%, and 67% amphotericin B resistance [8, 9]. Other NICUs report an emergent increase in the fluconazole-resistant *C. parapsilosis* causing NIC [10–12]. Fluconazole-resistant *C. parapsilosis* rates varied between 17% and 55% in studies from South Africa [10, 12]. A study from Taiwan reported an overall fluconazole resistance rate of 18.7% amongst *Candida* species causing NIC [11]. NeoOBS, a multi-country study to investigate severe neonatal bloodstream infections and clinical outcomes in LMICs, showed that among 127 neonates with NIC from 14 hospitals in 8 countries (2018–2021) the most common *Candida* species were *C. albicans* (35%), followed by *C. parapsilosis* (30%) and *C. auris* (14%) [5]. The overall resistance rate to fluconazole was 40%, 18% for amphotericin B and 0% for micafungin among the 103 isolates tested [5].

Studies from different regions in India show that *C. tropicalis* is and remains the most important species causing NIC followed by *C. parapsilosis*,* Pichia kudriavzevii* (formerly *Candida krusei*) and *Candida guillermondii* (Table 1) [13–22]. Of concern are the outbreaks of NIC due to rare yeast species such as *Candida utilis*,* Wickerhamomyces anomalus*,* Lodderomyces elongisporus*,* Candida blankii*,* C. auris*, and *P. kudriavzevii* (Table 2) [19, 23–29]. Importantly, these species are more often resistant to fluconazole and/or amphotericin B and occasionally to echinocandins.

**Table 1:** Epidemiology of neonatal candidemia in India

No.	Author, Year	State, Region	No. of isolates	Period	Species identified	Antifungal resistance
1	Metgud et al., 2024 [13]	Karnataka, South India	*n* = 16	Not given	*C. glabrata* (*n* = 8, 50%) *C. tropicalis* (*n* = 7, 43.8%) *C. krusei* (*n* = 1, 5.2%)	*C. tropicalis*: FLC R 42.8% 5FC R 28.6% AMB R 14.2%
2	Behera et al., 2023 [14]	Cuttack, Odisha	*n* = 124	2023	*C. tropicalis* (*n* = 36, 29.0%) *C. parapsilosis* (*n* = 31, 25%) *C. guilliermondii* (*n* = 17, 13.7%) *C. albicans* (*n* = 14, 11.3%) *C. utilis* (*n* = 12, 9.7%) *C. famata* (*n* = 7, 5.7%) *C. krusei* (*n* = 2, 1.6%) *C. glabrata* (*n* = 2, 1.6%) *C. kefyr* (*n* = 2, 1.6%) *C. lusitanae* (*n* = 1, 0.8%)	FLC R: *C. tropicalis* 44% *C. parapsilosis* 29.3%
3	Rai et al., 2024 [15]	Varanasi, Uttar Pradesh, North India	*n* = 107	2020–2022	*C. utilis* (*n* = 57, 53.3%) *C. pelliculosa* (*n* = 19, 17.7%) *C. tropicalis* (*n* = 9, 8.4%) *Kodamaea ohmeri* (*n* = 7, 6.5%) *Wickerhamomyces anomalus* (*n* = 6, 5.6%) *C. albicans* (*n* = 3) *C. glabrata* (*n* = 2) *C. orthopsilosis* (*n* = 2) *C. parapsilosis* (*n* = 1) *C. auris* (*n* = 1)	FLC R: *C. albicans* (2/3, 66.6%) *C. tropicalis* (1/9, 11.1%)
4	Biswas et al., 2023 [16]	Jharkhand, North eastern	*n* = 56	2020–2021	C. *tropicalis* (*n* = 26, 46.4%) *C. albicans* (*n* = 10, 17.9%) *C. parapsilosis* (*n* = 9, 16.1%) *C. gulliermondi* (*n* = 6, 10.7%) *C. krusei* (*n* = 5, 8.9%)	FLC R: *C. parapsilosis* 33.3% *C. krusei* 100% 5FC R: *C. krusei* 80% *C. tropicalis* 4%
5	Gautam et al., 2022 [17]	New Delhi, North India	116 of which 43.1% were neonatal candidemia (*n* = 50)	2019–2021	*C. tropicalis* (*n* = 31, 26.7%) *C. pelliculosa* (*n* = 23, 19.8%) *C. albicans* (*n* = 20, 17.2%) *C. parapsilosis* (*n* = 17,14.7%) *C. famata* (*n* = 11, 9.5%) *C. krusei* (*n* = 11, 9.5%)	*C. albicans*: VRC R 20% FLC R 5% *C. tropicalis*: VRC R 10.3% FLC R 6.9% CFG R 3.4% *C. parapsilosis*: CFG R 5.8% AMB R 5.8%
6	Kaur et al., 2021 [18]	Chandigarh, North India	*n* = 212	2017–2019	*W. anomalus* (*n* = 56, 26.4%) *C. utilis* (*n* = 52, 24.5%) *C. tropicalis* (*n* = 37, 17.5%) *C. krusei* (*n* = 18, 8.5%) *C.albicans* (*n* = 14, 6.6%) *C. parapsilosis* (*n* = 12, 5.7%) *C. glabrata* (*n* = 4, 1.9%)	Overall azole resistance 9.1%
7	Kaur et al., 2020 [19]	Chandigarh, North India	7927 of which 64.9% were neonatal candidemia (*n* = 5147)	1999–2018	*C. tropicalis* (36.5%) *W. anomalus* (18.8%) *C. albicans* (10.3%) *C. krusei* (8.1%) *C. gulliermondii* (5.3%) *C. utilis* (4.1%) *C. glabrata* (1.5%) *Kodamaea ohmeri* (1.5%) *C. parapsilosis* (1.4%)	Overall FLC R 7.4–8.8%
8	Nazir et al., 2018 [20]	Srinagar, Kashmir, North India	*n* = 80	2016	*C. tropicalis* (*n* = 34, 13.8%) *C. albicans* (*n* = 14, 5.6%) *C. krusei* (*n* = 12, 4.8%) *C. parapsilosis* (*n* = 8, 3.2%) *C. guilliermondii* (*n* = 7, 2.8%) *C. dubliniensis* (*n* = 5, 2.0%)	FLC R *C. albicans* 42% *C. tropicalis* 32.6%
9	Jajoo et al., 2018 [21]	New Delhi, India	*n* = 91	2011–2015	*C. tropicalis *(*n = *20, 22%) *C. albicans *(*n = *20, 22%) *C. parapsilosis *(*n = *18, 19.8%) *C. krusei *(*n = *16, 17.6%) *C. pelliculosa *(*n = *9, 9.9%) *C. glabrata *(*n = *8, 8.8%)	*C. krusei*: FLC R 92.9% VRC R 8.3% AMB R 14.3% *C. glabrata*: FLC R 87.5%
10	Lamba et al., 2021 [22]	Jaipur, Rajasthan (North Western)	*n* = 32	2014–2015	*C. tropicalis* (*n* = 14, 43.8%) *C. albicans* (*n* = 7, 21.9%) *C glabrata* (*n* = 6, 18.8%) *C. parapsilosis* (*n* = 4, 12.5%) *C. krusei* (*n* = 1, 3.1%)	FLC R: *C. krusei* 100% *C. albicans* 43% *C. glabrata* 33% *C. tropicalis* 7% CFG R: *C. albicans* 14%

*AMB* Amphotericin B; *CFG* Caspofungin; *5FC* Flucytosine; *FLC* Fluconazole; *VRC* Voriconazole

**Table 2:** Outbreaks of neonatal candidemia due to uncommon and rare yeasts in India

No	Author, year	State, region	No. of affected neonates	Outbreak duration	Species	Antifungal susceptibility	Risk factor
1.	Jain et al., 2025 [23]	Delhi, North India	*n* = 24	2 outbreaks during 2015–2020	*Pichia kudriavzevii* (formerly *C. krusei)*	Susceptible to ITC, VRC, PSC, AMB, MFG, AFG. Intrinsic resistance to FLC	Prematurity LBW Thrombocytopenia
2.	Singh et al., 2024 [24]	Uttar Pradesh, North India	*n* = 18	6 mo (2023–2024)	*Candida utilis*	Susceptible to FLC, VRC, ITC, AMB, 5FC	Extended ICU stay Prior antibiotic use
3.	Spruijtenburg et al., 2023 [25]	Chandigarh, North India	*n* = 29	10 mo (2019)	*Wickerhamomyces anomalus*	Resistance rates AMB 6.9% FLC 2.3% ITC 1.1% VRC 3.4%	
4.	Yadav et al., 2023 [26]	Delhi, North India	*n* = 10	6 mo (2021–2022)	*Lodderomyces elongisporus*	Susceptible to FLC, ITC, VRC, MFG, AFG, AMB, 5FC	Prematurity LBW
5.	Chowdhary et al., 2020 [27]	Delhi, North India	*n* = 9	7 mo (2016)	*Candida blankii*	High FLC (8 mg/ml) and AFG (2 mg/ml) MIC	Prematurity LBW Severe asphyxia
6.	Chowdhary et al., 2020 [28]	Delhi, North India	*n* = 12	7 mo (2016)	*Dirkmeia churashimaensis*	Susceptible to azoles, AMB and 5FC. Resistant to echinocandins.	Prematurity LBW Asphyxia
7.	Kaur et al., 2020 [19]	Chandigarh, North India	*n* = 65	1 y (2014)	*Candida krusei*	Susceptible to ITC, VRC, PSC, AMB and echinocandins. FLC not tested.	Congenital malformations ICU admission Mechanical ventilation
8.	Duggal et al., 2015 [29]	Delhi, North India	*n* = 13	3 outbreaks (2011, 2013 and 2014)	*Candida krusei*	Resistant to FLC. Susceptible to AMB, CFG, and MFG.	Prematurity LBW Asphyxia IUGR TPN

*AFG* Anidulafungin; *AMB* Amphotericin B; *CFG* Caspofungin; *5FC* Flucytosine; *FLC* Fluconazole; *ICU* Intensive care unit; *ITC* Itraconazole; *IUGR* Intra-uterine growth retardation; *LBW* Low birth weight; *MFG* Micafungin; *MIC* Minimal inhibitory concentration; *PSC* Posaconazole; *TPN* Total parental nutrition; *VRC* Voriconazole

## Management Approaches

To enable targeted treatment of NIC in an era of emerging resistance, identification to species level and antifungal susceptibility testing results are needed to choose the most appropriate antifungal agent. Nevertheless, surveys performed by the ECMM/ISHAM across hospitals in Europe, Africa and Asia/Pacific show poor availability of antifungal susceptibility testing for yeasts in Africa and Asia/Pacific (62% and 40% respectively) compared to Europe (93%) [30–32]. Consequently, NIC in LMICs will often be treated based on identification of isolated *Candida* sp., with the risk of choosing of an antifungal agent for which the *Candida* sp. is not susceptible. But even if antifungal susceptibility testing results are available, access to specific antifungal agents varies from continent to continent, and from country to country. Access to fluconazole across Europe, Africa and Asia/Pacific is > 90%, while access to micafungin is very limited in African hospitals (22.5%), and higher in those in Asia/Pacific (56%) and Europe (65%) [30–32]. Access to amphotericin B deoxycholate varies between 40% and 60% in these continents, while access to liposomal amphotericin B is very limited in African hospitals (17.5%), available in 57% of hospitals in Asia/Pacific, and in 78% of European hospitals. The limited availability and access to antifungal susceptibility testing and antifungal agents in LMICs are substantial, increasing the challenges to optimal management of NIC.

The recently published global guideline for the diagnosis and management of candidiasis recommends moderate strength amphotericin B deoxycholate, liposomal amphotericin B, micafungin and caspofungin for the treatment of NIC [33]. Fluconazole can be used in those neonates who have not been previously exposed to fluconazole and in settings with low fluconazole resistance (see Table 3 for recommended antifungal dosing in neonates). Neonatal invasive candidiasis is in up to 20% complicated by dissemination to the central nervous system, causing hematogenous meningoencephalitis (HCME). For HCME, micafungin is the echinocandin of choice, but a higher dose is recommended [33]. Based on the randomized controlled trials in cryptococcal meningitis, a recommendation for amphotericin B deoxycholate plus flucytosine in the treatment of HCME caused by susceptible *Candida* sp. can also be made. Taking into account the susceptibility profiles of both *C. auris* and *C. parapsilosis*, and the rare occurrence of echinocandin-resistant *Candida* spp. causing NIC, micafungin seems to be a good recommendation for empiric treatment when antifungal susceptibility results are not available. The WHO Essential Medicines List has added in the 2023 update micafungin in addition to fluconazole and amphotericin B for the treatment of invasive candidiasis [https://www.who.int/publications/i/item/WHO-MHP-HPS-EML-2021.02].

**Table 3:** Recommended dosing of antifungals in neonates [33]

Indication	Antifungal + dosing regimen
Prophylaxis	Fluconazole 3 mg/kg IV every 3rd day (wk 1–2), every other day (wk 3–4), daily (wk 5–6)
	Fluconazole 3 or 6 mg/kg IV/PO every other day for 6 wk
	Nystatin 100,000 IU 3 times/d PO
Treatment	Amphotericin B deoxycholate 0.7-1.0 mg/kg/d IV
	Fluconazole 25 mg/kg day 1, then 12 mg/kg/d IV
	Micafungin 15 mg/kg day 1, then 10 mg/kg/d IV
	Caspofungin 2 mg/kg/d IV
	Liposomal Amphotericin B 2.5-7.0 mg/kg/d IV
	Flucytosine 100–150 mg/kg d IV in 3–4 divided doses

*IV* Intravenous; *PO* Per os

The recommendations for neonatal antifungal prophylaxis are based on the epidemiology of NIC in HICs [33] and recent data indicate that these recommendations are not adequate for LMICs. In HICs, NIC is an infection affecting extreme premature neonates (< 28 wk gestation) and very-low-birth weight neonates (< 1000 g). In the NeoOBS study authors noticed that neonates developing NIC were less premature (median gestational age 30 wk) and had a higher median birth weight (1270 g). Only 19% and 27% had a gestational age < 28 wk and birth weight < 1000 g, respectively [5]. An epidemiological study of neonatal sepsis in India showed that 25% were caused by *Candida* spp. and 75% of the neonates were born > 32 wk of gestation and 60% had a birth weight of > 1500 g [21]. Neonatal antifungal prophylaxis should be targeted at the neonatal population most at risk with a relatively high incidence. Neonatal fluconazole prophylaxis has been well studied in various dose regimens and has shown to significantly decrease *Candida* colonization, *Candida* infection, and a statistically non-significant decrease in overall mortality in very-low-birth weight infants [34, 35]. Neonatal fluconazole prophylaxis is challenged by the increase in *C. auris* and fluconazole-resistant *C. parapsilosis* as the main causative species of NIC and is therefore not recommended in these settings. Oral nystatin is recommended as a good alternative [36], but unfortunately, *C. auris* has shown high resistance rates (52%-100%) against nystatin [37]. Another alternative is oral lactoferrin alone or combined with *Lactobacillus* [38] if available.

A decision to implement neonatal antifungal prophylaxis needs to be made carefully. Drawbacks of antifungal prophylaxis are consequences on the fungal epidemiology with more resistant species replacing susceptible species [39], potential adverse events and costs. Infection prevention and control measures are key and are the cornerstone of preventing neonatal infections including those caused by *Candida* spp. In a single centre study, fluconazole prophylaxis reduced the incidence of NIC from 22.3% to 18.2%, but adding a bundle of integrated IPC measures reduced the incidence to 2.9% [40].

## Infection Prevention and Control

Healthcare-associated neonatal infections and outbreaks have been proven more frequent in LMIC compared to HIC because of poor intrapartum and postnatal infection-control practices [41]. Different *Candida* spp. have been associated with neonatal outbreaks, especially in LMIC settings [27, 42, 43]. Fluconazole-resistant *C. parapsilosis* and *C. auris* have become increasingly involved in severe healthcare outbreaks in neonatal units [44–46]. This is facilitated by the capacity of these species to colonise human skin, disperse into the environment, survive on inanimate surfaces for extended periods, and resist routine disinfectants [47].

While there is limited evidence around infection prevention and control (IPC) interventions in neonatal units in LMIC [48]; these measures are crucial for preventing the spread of infections, including those caused by *Candida* spp. Infection prevention and control strategies encompass the standard NIC prevention measures, as well as those focused on the management of neonates infected or colonised by *C. auris*, or other fluconazole-resistant species, and the prevention of further transmission within the unit [49]. A summary of these measures is presented in Table 4.

The implementation of IPC measures for *Candida* infections in neonates presents several significant challenges. A major issue is the delayed identification of NIC which is complicated by its non-specific clinical presentation, the poor sensitivity of blood cultures, and limited access to rapid diagnostic tools. Resource limitations in LMICs settings exacerbate these challenges, with overcrowded neonatal units increasing the risk of environmental contamination [50], while staffing shortages might affect the adherence to IPC protocols and the ability to maintain a 1:1 patient-to-staff ratio, especially when *C. auris* cases are identified [49]. Furthermore, insufficient training and awareness among healthcare providers regarding *Candida* infections and IPC measures facilitate gaps in prevention strategies. Routine screening and surveillance for *Candida* colonisation are also often neglected due to logistical, financial, and technical constraints. Overcoming these barriers requires a multifaceted approach, including developing improved diagnostic tools, enhanced staff training programs, monitoring of compliance with IPC measures, and investment in infrastructure and resources to support effective IPC.

**Table 4:** Preventive measures targeted at resistant *Candida* species

**Standard prevention** These apply to all patients and in all situations and are designed to reduce the risk of transmission of microorganisms in healthcare settings.	
Hand Hygiene (HH)	Hand hygiene is a low-cost and efficient way to prevent infections in newborns. It offers an affordable and feasible solution for LMICs [41]. HH becomes essential for patient care after the isolation of a case of NIC, specially of *C. auris* or other resistant species in the unit [49]. HH can be improved by monitoring healthcare professionals combined with timely feedback [50].
Environmental Cleaning and Disinfection	Importance of routine cleaning and disinfection of NICU environments. In cases involving *C. auris*, it may be necessary to modify the choice of disinfectants. To ensure effective inactivation, peracetic acid (PAA)-based disinfectants are recommended over those containing quaternary ammonium compounds (QAC), with or without alcohol [49]. *C. auris* can contaminate healthcare environment rapidly after disinfection without appropriate products, as fast as 4 h [47].
Central Line-Associated Bloodstream Infection (CLABSI) Prevention	CLABSI prevention bundles have demonstrated significant efficacy in NICUs, achieving approximately a 60% reduction in infection rates following their implementation [51]. This is particularly relevant to *Candida* species, as evidence indicates that neonates with a *Candida*-colonised CVC present a ten-fold increased risk of developing NIC compared to colonised neonates without a colonised CVC [52].
Antibiotic Stewardship	Prolonged use of broad-spectrum antibiotics has been linked to the development of NIC, including among full-term infants [1]. Due to increasing antimicrobial resistances in LMICs, antibiotic regimens used in neonatal sepsis commonly diverge from WHO guidelines, increasing environmental pressure and risk of NIC [53].
Probiotics	While the use of single- and multi-strain probiotics, in combination with prebiotics or lactoferrin, has been shown to reduce neonatal morbidity and mortality, including the risk of late-onset sepsis [54]; this has not been proven for *Candida* infections and NIC.
Kangaroo Mother-Care	Although KMC has been found to reduce mortality and nosocomial infection/sepsis risk in low-birth-weight infants [55]; it’s applicability for NIC has not been elucidated.
Education and Training	Inadequate understanding of IPC measures, the role of personal protective equipment (PPE) and its suboptimal use can compromise its effectiveness, potentially failing to prevent or even increasing pathogen transmission during routine care or during infectious disease outbreaks.

**Measures after the identification of a case** Contact transmission-based precautions are designed to interrupt transmission of *C. auris* or other fluconazole-resistant *Candida* species, after the identification of a colonised or infected neonate.	
Isolate neonates infected and/or colonised	The isolation of the infected or colonised neonate in an individual space is crucial. If not possible, cohorting neonates is an alternative. Staff and parents should be educated on the importance of HH. In addition, contact precautions such as wearing gloves and aprons are recommended. Whereas patient isolation has been effective for *C. auris*, the recommendation for other fluconazole-resistant *Candida* infections is controversial and entails a logistical and economic burden, difficult to maintain in resource-limited settings [56]. Additionally, medical devices should be dedicated exclusively to the neonate and not shared with others [49].
Early Detection and Surveillance	In outbreak scenarios, it is critical to assess colonisation among epidemiologically linked neonates. Suitable specimens for analysis include axillary skin swabs, groin skin swabs, nasal or throat swabs, rectal swabs or stool samples, urine, wound exudate, and respiratory tract specimens. The axillae and groin areas have been identified as the most frequent and reliable sites for detection of colonisation [49].
Long term surveillance	Outbreaks of *C. auris*, fluconazole resistant *C. parapsilosis* or other resistant *Candida* species, are often prolonged, with new cases potentially emerging over several weeks or months. In the settings where this is possible, it is recommended that, even in the absence of additional cases, neonates within the affected unit undergo weekly screening using a combined groin-axilla swab (culture only) for a minimum of three months following the discharge of the last positive neonate [49].

*CVC* Central venous catheters; *IPC* Infection prevention and control; *LMICs* Low- and middle-income countries; *NIC* Neonatal invasive candidiasis; *NICU* Neonatal intensive care unit

## Summary

The fungal epidemiology of NIC has shown dramatic changes with non-*albicans Candida* species causing the vast majority of infections in neonates. In parallel, fluconazole-resistant NIC has progressively increased over the last decade, mainly affecting LMICs. This change in fungal epidemiology has challenged the management of NIC and pressurizes the few antifungal classes available for treatment. For prevention, IPC measures are as important, if not more, than antifungal prophylaxis, as the latter is associated with increased antifungal resistance. Neonates who might benefit from prophylaxis differ in clinical characteristics between HICs and LMICs, although current global management guidelines do not take this differentiation into account.
